# The importance of routine quality control for reproducible pulmonary measurements by in vivo micro-CT

**DOI:** 10.1038/s41598-022-13477-7

**Published:** 2022-06-11

**Authors:** Martina Mambrini, Laura Mecozzi, Erica Ferrini, Ludovica Leo, Davide Bernardi, Andrea Grandi, Nicola Sverzellati, Livia Ruffini, Mario Silva, Franco Fabio Stellari

**Affiliations:** 1 Department of Veterinary Science, University of Parma, Parma, Italy; 2 Department of Medicine and Surgery, University of Parma, Parma, Italy; 3 Pharmacology and Toxicology Department Corporate Pre-Clinical R&D, Chiesi Farmaceutici S.p.A., Largo Belloli, 11/A, 43122 Parma, Italy; 4 Division of Nuclear Medicine, Azienda Ospedaliero-Universitaria di Parma, Parma, Italy

**Keywords:** Quality control, Preclinical research, X-ray tomography

## Abstract

Micro-computed tomography (CT) imaging provides densitometric and functional assessment of lung diseases in animal models, playing a key role either in understanding disease progression or in drug discovery studies. The generation of reliable and reproducible experimental data is strictly dependent on a system’s stability. Quality controls (QC) are essential to monitor micro-CT performance but, although QC procedures are standardized and routinely employed in clinical practice, detailed guidelines for preclinical imaging are lacking. In this work, we propose a routine QC protocol for in vivo micro-CT, based on three commercial phantoms. To investigate the impact of a detected scanner drift on image post-processing, a retrospective analysis using twenty-two healthy mice was performed and lung density histograms used to compare the area under curve (AUC), the skewness and the kurtosis before and after the drift. As expected, statistically significant differences were found for all the selected parameters [AUC 532 ± 31 vs. 420 ± 38 (p < 0.001); skewness 2.3 ± 0.1 vs. 2.5 ± 0.1 (p < 0.001) and kurtosis 4.2 ± 0.3 vs. 5.1 ± 0.5 (p < 0.001)], confirming the importance of the designed QC procedure to obtain a reliable longitudinal quantification of disease progression and drug efficacy evaluation.

## Introduction

Computed tomography (CT) represents one of the most relevant imaging technologies for the assessment of lung disorders in clinical practice. Since lung densitometry depends on X-ray (XR) attenuation of pulmonary tissue, changes in lung tissue density can reflect parenchymal abnormalities providing a reproducible quantitative evaluation of the extent and severity of pulmonary diseases^1–3^*.*

The miniaturized version of CT (i.e. micro-CT) represents a powerful non-invasive tool in preclinical research for understanding the pathogenesis and dynamics of lung disorders in several animal models^4–8^.

Histogram-based analyses are used to discriminate between aeration levels, identifying variations in lung density to allow accurate longitudinal assessment of lung disease progression^8,9^ in compliance with the 3R rules (Replacement, Reduction, Refinement). Moreover, the integration of micro-CT technology in antifibrotic drug discovery can be useful for investigating drug efficacy in preclinical settings^10–13^*.* However, timing, reliability and reproducibility of the data generated by micro-CT in preclinical studies present crucial issues in characterizing the human disease and profiling the putative drug candidate for a move into the clinic. Although standardized protocols for image acquisition and anesthesia are adopted to generate accurate and reproducible datasets^14^, other factors can potentially undermine either the intra- or the inter-experiment reproducibility of results.

The implementation of quality control (QC) protocols is therefore essential in order to constantly monitor scanner performance: scan quality may be altered by instrumental drift, thus affecting image post-processing^15^ and the interpretation of CT results, especially when a fully automatic analysis of CT scans is used^16^.

Standardized quality procedures are employed for clinical CT systems to optimize patient safety and the reliability of densitometric data, to ascertain that image quality requirements are met and to prevent system drift over time. According to clinical requirements, daily and monthly QC protocols are applied based on dedicated CT phantoms^17–19^, beyond the requirements of annual maintenance^20–24^.

In contrast, only general guidelines are available for preclinical micro-CT scanners^25^, which leave aside longitudinal monitoring and focus on validating home-made CT imaging systems^26^ or characterizing their performance in terms of image quality, stability and X-ray delivered dose during acquisition^27–29^. Additionally, in-house-developed devices and protocols^15,30,31^ are not easily applicable to other imaging centres.

In this work, we outline a routine QC protocol for in vivo micro-CT, based on commercial preclinical phantoms, that is able to monitor over months the stability of the scanner to detect any drift and ensure reproducible longitudinal pulmonary measurement. The designed QC procedure is intended to be a detailed guideline, adaptable in its implementation for any micro-CT scanner.

## Materials and methods

Ad-hoc QC tests for micro-CT systems have been established, using three commercial imaging phantoms (QRM GmbH, Möhrendorf, Germany) (Table 1).

**Table 1:**
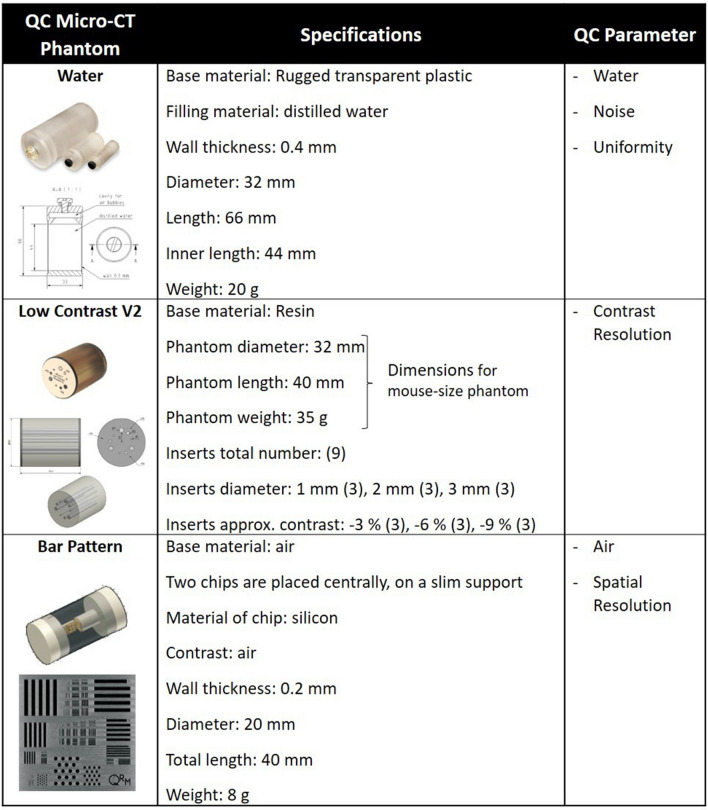
QC phantom specifications as reported in the manufacturer’s datasheets (QRM GmbH, Möhrendorf, Germany) and corresponding QC parameters for which they are used.

**Figure 1:**
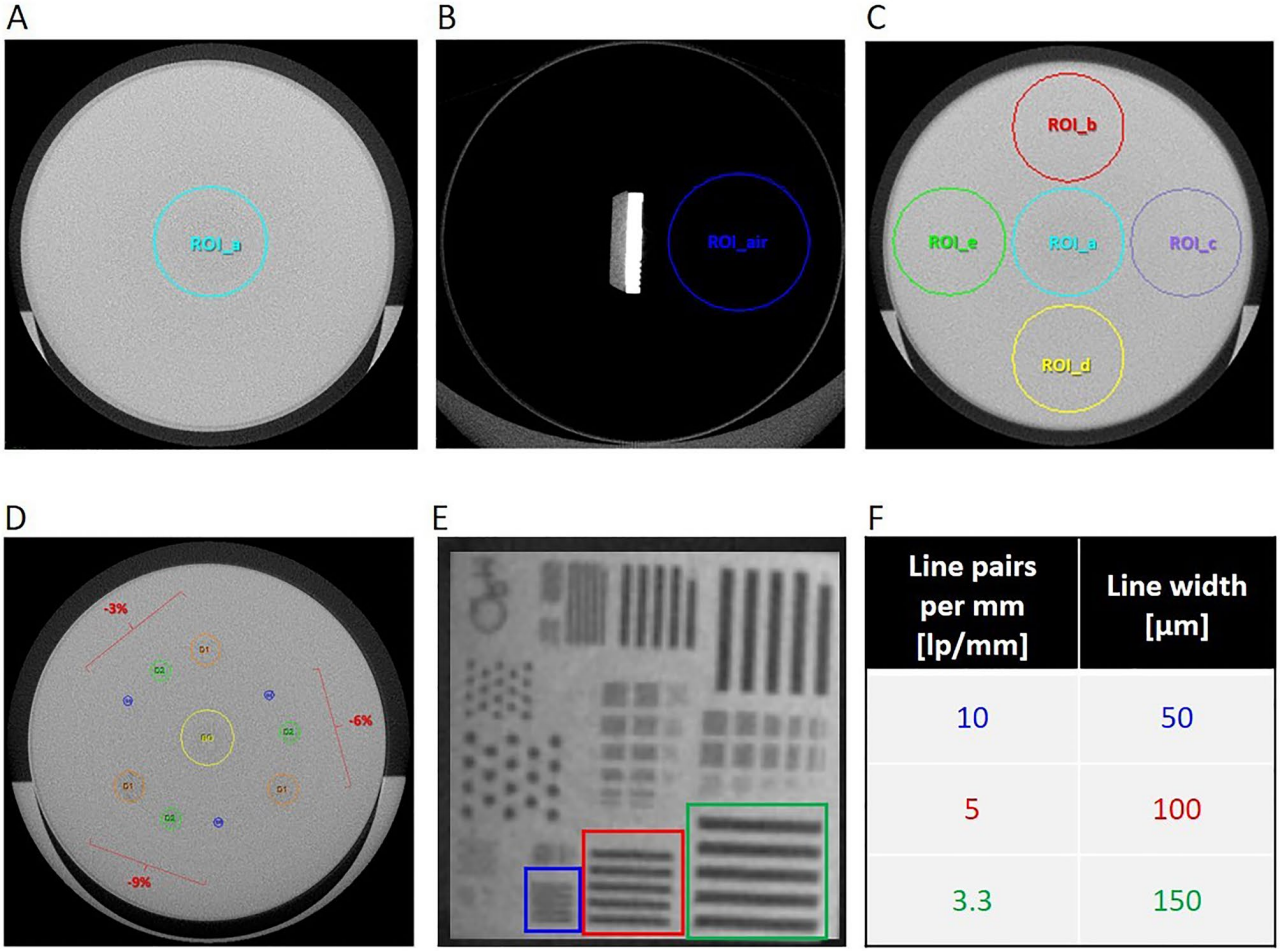
Axial views of micro-CT phantoms and segmentation maps used for the corresponding QC tests. (**A**) Water phantom with the ROI used to perform noise test and water value evaluation, within five contiguous slices. (**B**) Bar Pattern phantom filled with air with the ROI used to monitor air value, within five contiguous slices. (**C**) Water phantom with the ROIs used for the uniformity test, within five contiguous slices: one central (ROI_a) and four peripheral ROIs (ROI_b, ROI_c, ROI_d, ROI_e). (**D**) Low Contrast V2 phantom: the cylindric inserts with different low contrast levels (− 9, − 6 and − 3%) are identified by means of proportional ROIs, orange for 3 mm diameter, green for 2 mm diameter and blue for 1 mm diameter. The central yellow ROI is used to detect the background value. (**E**) The 5 × 5 mm^2^ chip of the Bar Pattern phantom: the patterns used for the spatial resolution test are indicated in blue (10 lp/mm), red (5 lp/mm) and green (3.3 lp/mm) and (**F**) the corresponding line widths are reported.

Phantoms are acquired by micro-CT monthly^25–28,31^ and segmentation maps with fixed regions of interest (ROIs) are applied to the scans for the assessment of the image quality parameters (Fig. 1). These measurements are conducted in a grey levels scale, that corresponds to the original scale of CT scans after reconstruction.

The data reported in this study were collected on specific control charts and compared to baseline (BL) values, each defined by upper and lower tolerance limits, as suggested by literature^25,31^. BL values were initially established for our Quantum GX micro-CT (PerkinElmer, Inc. Waltham, MA), acquiring five consecutive scans of the QC phantoms^31,32^. For each monitored parameter, the BL and tolerance width were calculated, respectively:

$$ BL = Average\left( {\left( {x_{j} } \right)_{j = 1, \ldots ,5} } \right), $$

$$ Tolerance\,width = 2 \times SD\left( {\left( {x_{j} } \right)_{j = 1, \ldots ,5} } \right), $$

in which $x$ represents an image quality parameter, $j$ is the index of weekly acquisition and SD is the standard deviation of the baseline measures. All measures obtained by the QC tests were then compared to the corresponding tolerance range (Table 2):

$$ Tolerance\,range = BL \pm Tolerance\,width. $$

**Table 2:** Tolerance ranges calculated for all the monitored QC parameters for the Quantum GX micro-CT (PerkinElmer, Inc. Waltham, MA).

QC parameter	Tolerance range (BL ± tolerance width)
Water (grey level)	2000 ± 17
Air (grey level)	449 ± 33
Noise (grey level)	124 ± 2
Uniformity (grey level)	− 41 ± 22
Contrast Resolution (%)	− 9%: − 8 ± 3
	− 6%: − 3 ± 1
	− 3%: 1 ± 1
MTF (%) for spatial resolution	3.3 lp/mm: 49 ± 19
	5 lp/mm: 42 ± 17
	10 lp/mm: 24 ± 14

In this study, the dosimetry of the Quantum GX scanner^33^ and its impact on lung imaging were not objects of investigation, since they have been already evaluated in preclinical applications^34–36^, with findings suggesting that animal welfare was protected in this respect and that longitudinal CT data were not affected by multiple X-ray expositions.

### Micro-CT phantoms and corresponding QC tests

Water, Low Contrast and Bar Pattern phantoms (QRM GmbH, Möhrendorf, Germany) were used for quality control tests, as detailed below.

#### Water phantom

A cylindric device with a diameter of 32 mm and filled with milli-Q water, was employed to evaluate noise, to extract the absolute grey value for water and measure uniformity (micro-CT water phantom, QRM GmbH, Möhrendorf, Germany).

##### Noise test

The noise value was extracted applying a circular ROI (ROI_Noise_) to five spatially contiguous reconstructed slices, along the longitudinal z-axis (Fig. 1A). The ROI area (80.4 mm^2^) was chosen to be 10% of the cylinder base area (16 mm × 16 mm × π = 804 mm^2^), according to IPEM and IEC indications^37,38^. The noise was defined as the SD of the water grey level (i.e. SD^water^) within the ROI (i.e. mean^water^ ± SD^water^). In order to obtain the monthly noise value, namely ${Noise}$, we calculated the average of the five SD^water^:

$$ Noise = \mathop \sum \limits_{i = 1}^{5} \frac{{\left( {SD_{i}^{water} } \right)}}{5}\quad {\text{i }} = {\text{ slice}}\,{\text{index}}{.} $$

##### Water evaluation

Water grey level was monitored applying the same ROI employed for the noise test (Fig. 1A). In this case, we evaluated the mean water grey level value (mean_water_), averaging the values of the five contiguous cross sections that compose the ROIs:

$$ Water = \mathop \sum \limits_{i = 1}^{5} \frac{{\left( {mean \,value_{i}^{water} } \right)}}{5}\quad {\text{i }} = {\text{ slice}}\,{\text{index}}{.} $$

##### Uniformity test

Five circular ROIs were positioned in the centre and in peripheral locations of the image and propagated for five contiguous slices along z-axis, as shown in Fig. 1B. The image quality parameter for the uniformity test, i.e. $Uniformity$, was calculated as the difference between the mean^water^ value of the central ROI_a_ and the average of the four mean^water^ values of the peripheral ROIs (ROI_b_, ROI_c_, ROI_d_, ROI_e_):

$$ Uniformity = mean\,value_{{ROI_{a} }} - \mathop \sum \limits_{h = b}^{e} \frac{{\left( {mean\,value_{{ROI_{h} }} } \right)}}{5}\quad {\text{h }} = {\text{ ROI}}\,{\text{index}}{.} $$

Each ${mean value}_{ROI}$ was calculated by averaging the mean of water grey values obtained from the five contiguous slices, as in the *‘Water evaluation’* method.

#### Micro-CT low contrast phantom

The low contrast phantom was chosen to measure the contrast resolution of the scanner. It provides three approximate contrast levels of − 9%, − 6% and − 3% compared to the background material, each level composed of three circular inserts with different diameters, i.e. 1, 2 and 3 mm, for a total of nine inserts (micro-CT Low Contrast Phantom V2, QRM GmbH, Möhrendorf).

##### Low contrast test

The low contrast detectability (LCD) is defined as the minimum visible dimension for low contrast objects, provided by the diameter of the smallest circular insert. The segmentation map for the LCD test consists of nine circular ROIs with dimensions proportional to the three different sizes of the inserts (Fig. 1D). After adjusting the position of the ROIs following the location of the inserts, the nine ROIs were propagated on 100 image slices to obtain a 3D evaluation. Two additional circular ROIs were also considered: one for resin background and one for air (outside the phantom). The image quality parameter for the LCD test, called $Contrast$ and expressed in %, was calculated as follows:

$$ Contrast_{k} = \left[ {1 - \frac{{\left( {mean\,value_{k} - mean\,value_{air} } \right)}}{{\left( {mean\,value_{background} - mean\,value_{air} } \right)}}} \right] \times 100, $$

where k represents the insert index and ranges from one to nine (k = 1,…,9). Finally, for each contrast level (− 9%, − 6%, − 3%), the $Contrast$ parameter was evaluated as the average value of the three inserts with the same low contrast level but different diameter sizes:

$${Contrast}^{c}=\frac{\left({Contrast}_{1mm}^{c}+{Contrast}_{2mm}^{c}+{Contrast}_{3mm}^{c}\right)}{3}$$

in which the index *c* can be − 9%, − 6% or − 3%. In this way, each month we obtained three values of $Contrast$, one for each nominal level.

#### Micro-CT bar pattern phantom

The spatial resolution test, as well as the evaluation of the absolute grey value for air, were carried out with the bar pattern phantom filled with air, and with a diameter of 20 mm. Two chips placed inside the phantom contain bar patterns with different widths and points with different diameters allowed evaluation of the spatial resolution in the centre as well as in the periphery of the image in a single measurement (micro-CT Bar Pattern Phantom, QRM GmbH, Möhrendorf).

##### Spatial resolution test

The in-plane spatial resolution for micro-CT images was tested. Based on visual inspection of the line pairs in the phantom scan (Fig. 1E), the spatial resolution was in a range between 10 and 3.3 line pairs per mm (l p/mm), corresponding to 50–150 µm line width (Fig. 1F), which represents the range in which the bars can be resolved^26^. The Modulation Transfer Function (MTF) of a system is used to describe the resolution performance of the scanner, providing a quantitative measure of the relationship between the original object and its radiological reconstruction^39^. MTF was calculated monthly for the three different bar patterns (3.3 lp/mm, 5 lp/mm and 10 lp/mm) using the ‘line profile’ tool of Analyze software (Analyze 12.0; Copyright 1986–2017, Biomedical Imaging Resource, Mayo Clinic, Rochester, MN), https://www.analyzedirect.com. The value averaged over three measures for ‘background’ and ‘bars’ was recorded and MTF calculated as follow:

$$ MTF_{{}}^{c} = \left[ {\frac{{\left( {mean\,value_{background} - mean\,value_{bars} } \right)}}{{\left( {mean\,value_{background} + mean \,value_{bars} } \right)}}} \right] \times 100, $$

where the index c can be 3.3 lp/mm, 5 lp/mm or 10 lp/mm. Since axial resolution is the same as transversal resolution, only the latter data are reported.

##### Air evaluation

The monitoring of grey levels of air was performed using a circular ROI positioned inside the bar pattern phantom (in air), with an area of 31.4 mm^2^, thus covering 10% of the phantom section area (10 mm × 10 mm × π = 314 mm^2^). The ROI was propagated for five contiguous cross sections and the mean grey level of air (mean_air_) was calculated as the average of the five values extracted, as following:

$$ Air = \mathop \sum \limits_{i = 1}^{5} \frac{{\left( {mean\,value_{i}^{air} } \right)}}{5}\quad {\text{i }} = {\text{ slice}}\,{\text{index}}{.} $$

### Experimental control animals

A cohort of 22 control mice were used for a retrospective evaluation of the impact of routine QC tests on lung scans post-processing.

Female inbred C57Bl/6 (7- to 8-weeks old) mice were purchased from Envigo, Italy (San Pietro al Natisone, Udine, Italy). Prior to use, animals were acclimatized for at least 5 days to the local vivarium conditions (room temperature: 20–24 °C; relative humidity: 40–70%; 12-h light–dark cycle), having free access to standard rodent chow and softened tap water.

All animal experiments described herein were authorized by the official competent authority and approved by the intramural animal-welfare body (AWB) of Chiesi Farmaceutici and authorized by the Italian Ministry of Health (protocol number: 809/2020-PR). All procedures were conducted in an AAALAC (Association for Assessment and Accreditation for Laboratory Animal Care) certified facility in compliance with the European Directive 2010/63 UE, Italian D.Lgs 26/2014, the revised “Guide for the Care and Use of Laboratory Animals”^40^ and with the Animal Research: Reporting of In Vivo Experiments (ARRIVE) guidelines^41^.

Animals were lightly anesthetized with 2.5% isoflurane delivered in a box and vehicle (50 μl saline [0.9%]) was administered via oropharyngeal aspiration (OA) using a micropipette^42^. Twenty-one days after saline administration, mice were anesthetized with 2% isoflurane and underwent micro-CT imaging^10,14^.

### Micro-CT settings, images acquisition and processing

The Quantum GX Micro-CT (PerkinElmer, Inc. Waltham, MA) was used in this study. This scanner has a microfocus X-ray source with a Tungsten anode. A fixed filter of 0.5 mm aluminium (Al) and 0.06 mm copper (Cu) is placed in front of the exit port to remove low energy X-rays. QC phantoms were acquired with the following parameters: X-ray tube voltage 90 kV, X-ray tube current 88 μA, total scan time of 4 min. A sinogram-based ring reduction filter was used to minimize rings inherent in CT scans. After the ring reduction was applied to the sinograms, the resulting corrected sinograms were input to the GPU-based filtered back-projection algorithm with a Ram-Lak filter. The acquisition protocol allows acquisition of projections over a total angle of 360° resulting in 3D datasets with 50 μm isotropic reconstructed voxel size^10,33^. All the images were imported as 3D .vox files and analysed using Analyze software (Analyze 12.0; Copyright 1986–2017, Biomedical Imaging Resource, Mayo Clinic, Rochester, MN) https://www.analyzedirect.com.

All protocols for mouse lung acquisition and image post-processing were largely detailed by Mecozzi et al.^10^ applied to a bleomycin-induced fibrosis murine model. Lung density histograms were extracted from pulmonary scans and the area under curve (AUC), the skewness and kurtosis calculated.

### Statistical analysis

Statistical analyses were performed using GraphPad Prism version 9.1.2 for Windows (GraphPad Software, La Jolla, CA, USA), https://www.graphpad.com. All data were presented as mean ± SD. The normality test was performed for the AUC, the skewness and kurtosis of the average lung histograms. A one-tailed unpaired t-test was performed to compare AUC parameters. Finally, an unpaired t-test and Mann–Whitney test were performed to compare the kurtosis and the skewness, respectively. The alpha level of all tests was set at 0.05.

## Results

**Figure 2:**
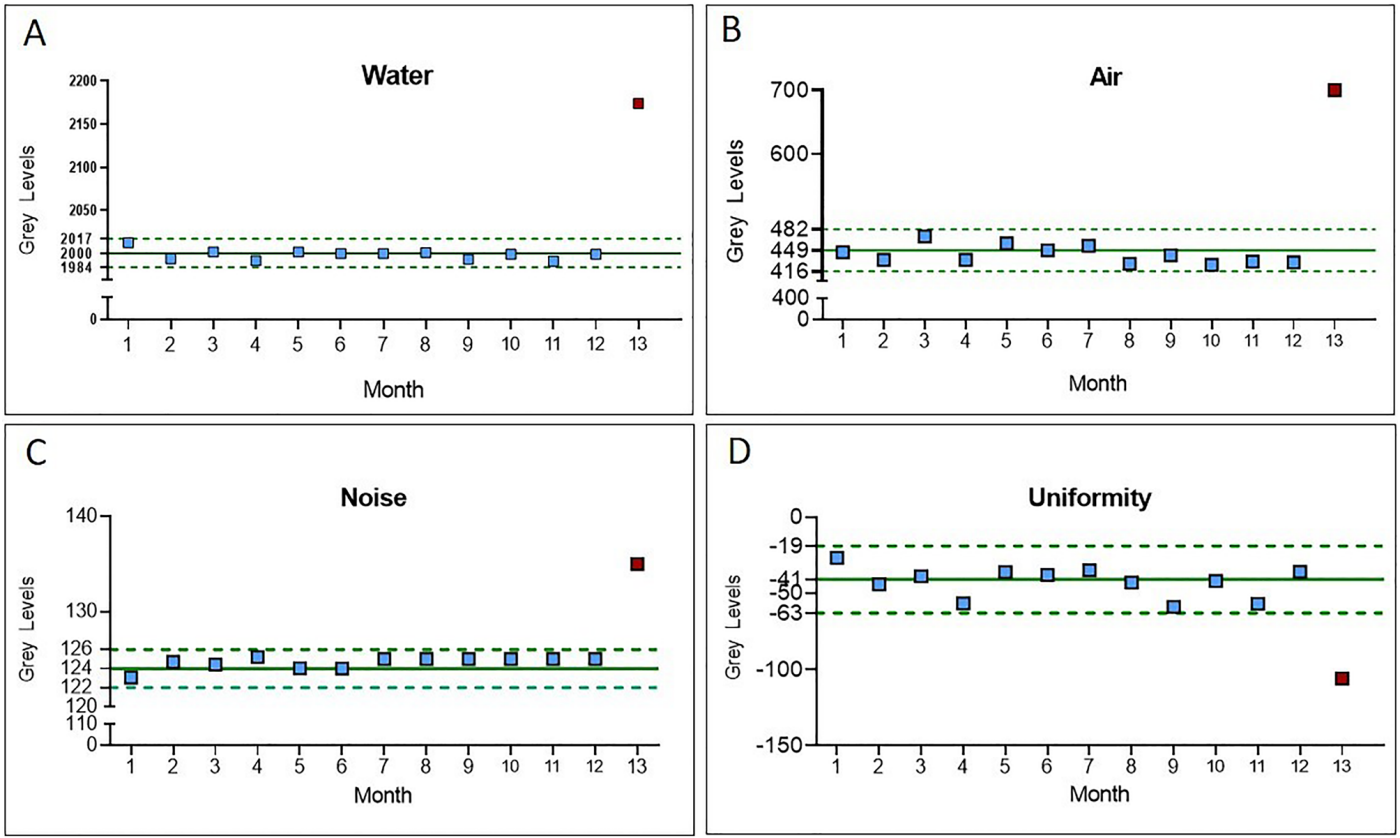
Control charts for Water and Air absolute grey levels (**A**,**B**) and Image noise and uniformity (**C**,**D**). The fluctuations of QC parameters were monitored during 13 months. BL values (continuous lines) and acceptability ranges (dotted lines) are highlighted in green for each control chart.

The grey level values of the QC parameters, monitored over 13 months, are reported in the control charts (Fig. 2). For each test, BL appears as a continuous line, while dotted lines correspond to the upper and lower limits of the tolerance range.

The grey levels values for water (Fig. 2A) and air (Fig. 2B) remained stable within their tolerance ranges until the 12th month (blue squares). In the 13th month a shift towards higher levels was observed (red square), out of the upper limit of the tolerance ranges (2174 and 700 for water and air, respectively).

Also, noise and uniformity values (Fig. 2C,D, respectively) were subjected to an analogous deviation from the BL values in the 13th month; they reached 135 and − 106 grey levels, respectively, thus deviating from the established tolerance ranges for noise (122, 126) and uniformity (− 63, − 19).

**Figure 3:**
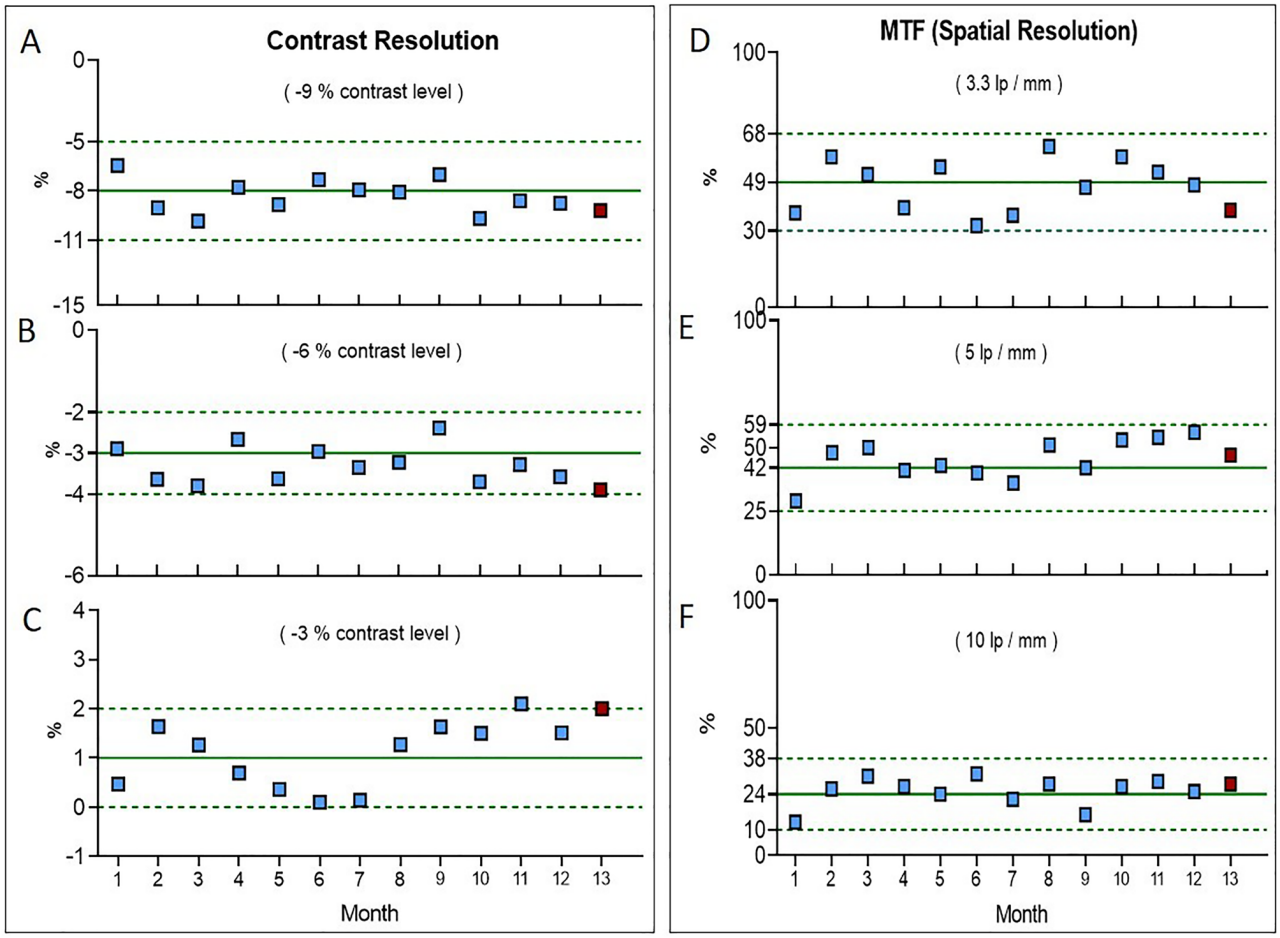
(**A**–**C**) Low contrast control charts for the known contrast levels of − 9%, − 6% and − 3%, respectively. (**D–F**) Spatial resolution control charts, in terms of MTF%, for the three different line pairs per mm patterns: 3.3 lp/mm, 5 lp/mm and 10 lp/mm, respectively. The fluctuations of the QC parameters were monitored over 13 months. BL values (continuous lines) and acceptability ranges (dotted lines) are reported in green for each control chart.

Figure 3A–C, instead, shows the trends observed for the nominal contrast levels − 9%, − 6% and − 3%. The BL values, measured for each contrast level, are different from the corresponding nominal value previously defined. We initially evaluated this discrepancy and agreed that it was acceptable for our purpose. Although the chosen acceptability ranges are narrow, the measurements performed in the 13th month were inside the tolerance limits for all the contrast levels. This means that during the whole observation period, the scanner was able to detect low contrast levels in lung scans, such as the tissue-air contrast^26^, despite the drift observed in the other parameters. The MTF for each of the three selected lp/mm bar patterns (i.e. spatial frequency) is reported in Fig. 3D–F and expressed as a percentage (%). These remained almost stable over the 13 months of evaluation and, overall, within acceptable values.

As a whole, as clearly depicted in Fig. 2, the last point of observation (red square) in the control charts represents an out-of-control point for the majority of QC parameters: an increase in image noise, a decrease in image uniformity, along with strong fluctuations of water and air values towards higher grey levels.

To better investigate the effect of this scanner’s drift on pulmonary scan post-processing and lung aeration levels, we performed a retrospective analysis on scans from 22 saline-treated mice, used as negative controls in lung fibrosis animal models. These animals were imaged during the period of investigation before (1st–12th months) and after the scanner’s drift (13th month) and were included in the analysis.

**Figure 4:**
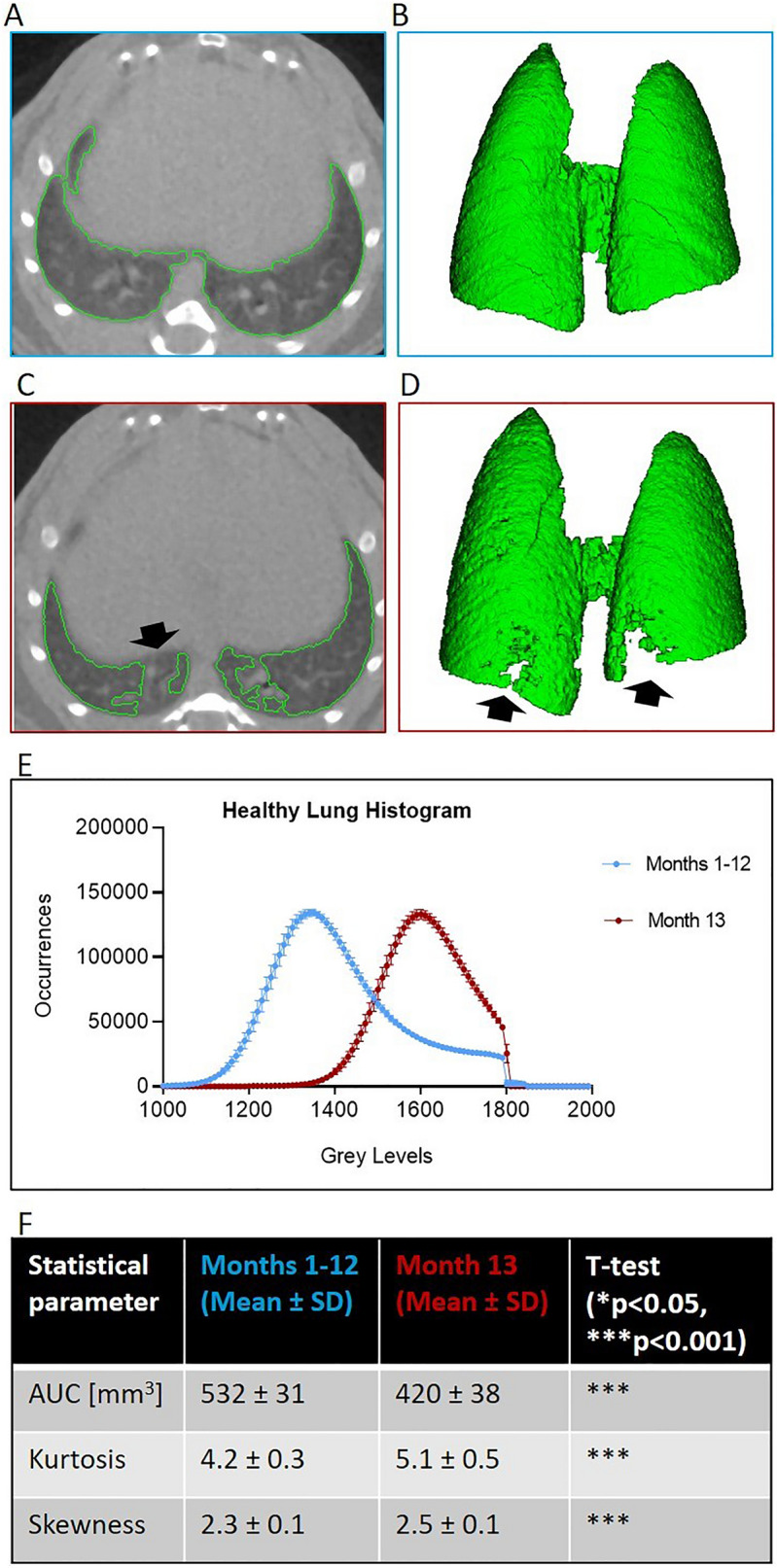
Effect of micro-CT scanner drift on image lung segmentation process. (**A**,**B**) A representative transversal slice of a saline mouse lung scan before the drift (1st–12th months): the lung segmentation map and the corresponding 3D rendering, obtained using an automatic segmentation, are highlighted in green. (**C**,**D**) A representative transversal slice and the corresponding 3D rendering of a saline mouse lung scan from the 13th month: the automatic segmentation fails to detect some healthy regions of aerated parenchyma (black arrows). (**E**) Lung mean grey levels density histograms before (1st–12th month: blue curve) and after (13th month: red curve) drift. Each point of the histogram is represented as mean ± SD. (**F**) Statistical comparison between the two average histograms in terms of AUC, Kurtosis and Skewness. All parameters are reported as mean ± SD. The alpha level of statistical tests is set at 0.05 (*p < 0.05, **p < 0.01, ***p < 0.001).

The lungs of saline-treated mice are characterized by homogenous and well-aerated parenchyma. As expected, in the first period of observation, the lungs were entirely detectable and extracted using a semi-automatic segmentation protocol with well-defined thresholds^10^, as shown in Fig. 4A (segmentation map) and Fig. 4B (lung volume rendering). After the scanner drift, however, although experimental conditions, acquisition and image analysis protocols were unchanged, the usual segmentation protocol failed to detect the whole healthy parenchyma (Fig. 4C,D). As outlined in the three-dimensional rendering (Fig. 4D) some regions were excluded from the segmented map, even if they were sufficiently aerated to be detected and segmented.

We also extracted the density histograms of the healthy lungs to assess the impact of the scanner’s drift on image analysis. Figure 4E shows the average histograms, regarding the two periods before (1st–12th month: blue curve) and after (13th month: red curve) drift. The blue and red curves are averaged over 12 and 10 subjects, respectively. The shift of the red histogram towards higher grey levels could be linked to the concomitant increase in grey levels of water and air in QC scans detected during the 13th month. Using the established thresholds to segment lungs, we would wrongly generate incomplete lung maps, as detailed in Fig. 4F. The AUC of the red histogram, which represents the total lung volume was, in fact, significantly smaller than the AUC of the blue histogram (532 ± 31 blue lung volume vs. 420 ± 38 red lung volume; p < 0.001). Moreover, both skewness (2.3 ± 0.1 blue vs. 2.5 ± 0.1 red; p < 0.001) and kurtosis (4.2 ± 0.3 blue vs. 5.1 ± 0.5 red; p < 0.001) parameters showed statistically significant differences, suggesting unjustified changes in the shape of the curves and the lung volumes of these healthy mice.

## Discussion and conclusion

In this work we demonstrated that changes in a micro-CT system can affect the quantitative analysis of CT images. Thus, a constant monitoring of micro-CT scanner performance is fundamental to recognizing instrumental drifts that can alter scan quality, impair image post-processing^15^ and corrupt the interpretation of CT results.

Although consistent guidelines for preclinical QC in micro-CT systems are still lacking, the definition of QC parameters and their tolerance ranges is a preliminary and very crucial step for the routine monitoring of such systems’ performance. Starting from commercial QC phantoms we provided a standardized protocol for phantom acquisition and analysis. Contrary to clinical indications, which specifically define BL ± 20% as the tolerance range for noise parameter, we found it more appropriate to consider BL ± 2 × SD as the tolerance range for noise evaluation, as well as for the other QC parameters. This allowed identification of relevant fluctuations in some QC parameters, such as noise, uniformity and water/air grey levels, occurring as a result of a drift in the instrument.

We also demonstrated the importance of measuring the absolute grey levels of water and air during QC procedures, with the lung parenchyma being a soft tissue composed of tissue and air, and the latter representing its natural contrast. These two parameters have proven useful in detecting the shift in terms of grey levels which can affect micro-CT analysis^15,30^. In clinical practice, only Nowik et al.^32^ have suggested a protocol capable of monitoring the value of air during QC acquisition, but putting the ROI outside the phantom (contrary to the IEC recommendations), thus resulting in a highly variable estimation.

As a whole, different scenarios could be possible depending on the factors impacting on QC parameters and on their mutual influence.

In any CT system, the image noise and blurring place upper limits on the spatial resolution and their relative importance is a function of image contrast. Moreover, the visibility of low contrast objects is forced mainly by the image noise characteristics. A reduction in tube load, being proportional to the photon fluence, will cause an increase in the image noise because of fewer photons contributing with information to the image. Changes in tube voltage will result in a change in image noise and contrast, as the voltage determines the energy of the photons emitted from the X-ray tube: the noise change is approximately inversely proportional to the change in tube voltage, but increasing the voltage can (slightly) reduce subject contrast as well^43^. The deterioration of the X-ray tube or the artifacts resulting from imperfections in scanner function (e.g. detectors out of calibration) could also lead to a systematic increase in noise and decrease uniformity^31,44^. Furthermore, X-ray filtration, helping in absorbing the photons that do not have enough energy to penetrate the body and reach the detectors, provides better noise and uniformity within the image and reduces the degradation of the image improving resolution.

In this work, the variations we detected in the QC parameters acquired in the 13th month of observation suggested the need for a technical intervention. Probably, the worsening of the image quality, due to the increase of noise and the decrease of uniformity was caused by the deterioration of the aluminium-copper filter placed in front of the exit port of the source^32^. Among the parameters considered, these factors can directly influence the voxel value, the parameters related to the X-ray tube output and the reconstruction phase.

The retrospective analysis on data from healthy lungs confirmed that alterations in the micro-CT system (e.g. CT scanner reconstruction’s algorithm and calibration, X-ray tube output, possible artefacts, etc.) impaired the post-processing phase. In pulmonary preclinical research, minor changes, if not immediately identified, negatively influence the lung segmentation process and the following densitometric quantification of disease progression or drug efficacy evaluation. For this reason, the ongoing monitoring of QC parameters is essential to ensure robust and reliable results during longitudinal studies, when the same animal is scanned weekly by micro-CT.

In addition, since the timing and the huge number of animals used in preclinical studies are leading to the integration of Artificial Intelligence models in pursuit of a completely standalone segmentation process^45^, the implementation of precise quality control procedures become mandatory.

To conclude, in this work we present a feasible and detailed operator-independent procedure for Quality Control of a micro-CT scanner, based on the acquisition of commercial QC phantoms, which any imaging facility could potentially integrate into their routine activities.
